# Role of Echocardiography in Detecting Left Ventricular Dysfunction Among Diabetic Patients: A Clinical and Biochemical Perspective

**DOI:** 10.7759/cureus.78720

**Published:** 2025-02-07

**Authors:** Chandu Siripuram, K. Balu Mahendran, Shreelaxmi V Hegde, Sanjana Murali Krishna, Shruti Suresh Suvarna, Ramesh Kandimalla

**Affiliations:** 1 Department of Community Medicine, Geisinger Community Medical Center, Scranton, USA; 2 Department of Biochemistry, Siddhartha Medical College, Vijayawada, IND; 3 Department of Biochemistry, Srinivas Institute of Medical Sciences and Research Center, Rajiv Gandhi University of Health Sciences, Mangalore, IND; 4 Department of Community Health Sciences, American University of Barbados, Wildey, BRB; 5 Department of Biochemistry, Government Medical College Narsampet, Sarwapuram, IND

**Keywords:** cardiovascular risk, diabetes mellitus, echocardiography, glycemic control, left ventricular dysfunction, systemic inflammation

## Abstract

Background: Diabetes mellitus (DM) increases the risk of left ventricular dysfunction (LVD), which can progress to heart failure if undetected. Echocardiography, a non-invasive and cost-effective imaging tool, provides real-time assessment of left ventricular (LV) function and enables early detection of myocardial dysfunction using advanced techniques such as tissue Doppler imaging and strain analysis. Diabetic patients are particularly prone to LVD due to chronic hyperglycemia, insulin resistance, and systemic inflammation, leading to myocardial fibrosis, microvascular dysfunction, and oxidative stress. This study evaluates the role of echocardiography in detecting subclinical and overt LVD in diabetic patients and explores associated clinical and biochemical risk factors.

Methods: This observational cohort study included 500 diabetic patients aged 30-70 years, with the sample size determined using power calculation (95% confidence level, 5% margin of error). Stratified random sampling was used for participant selection, with hospital-based recruitment noted as a limitation. Patients underwent clinical evaluation, biochemical analysis, and echocardiographic assessment, including left ventricular ejection fraction (LVEF), diastolic function indicators, and LV mass index. Biochemical markers analyzed included fasting blood glucose, HbA1c, lipid profile, and high-sensitivity C-reactive protein (hs-CRP), which was selected for its strong association with cardiovascular risk and myocardial dysfunction. Data analysis was performed using IBM SPSS Statistics for Windows, Version 26 (Released 2019; IBM Corp., Armonk, New York, United States), applying descriptive statistics, correlation studies, and multivariate logistic regression, with adjustments for age, gender, BMI, hypertension, and diabetes duration.

Results: Among the 500 participants, 140 (28%) exhibited diastolic dysfunction, while 90 (18%) had reduced LVEF (<50%), indicating diastolic dysfunction as the predominant abnormality. Patients with LVD had worse glycemic control and higher systemic inflammation markers than those with normal LV function. hs-CRP negatively correlated with the LVEF (r = -0.34, p = 0.022) and positively with the LV mass index (r = 0.38, p < 0.05), highlighting its role in myocardial remodeling. Multivariate analysis identified poor glycemic control and systemic inflammation as key predictors of LVD. Subgroup analysis showed that older patients (≥60 years) and those with diabetes duration >10 years had a higher prevalence of diastolic dysfunction and an increased LV mass index, suggesting progressive myocardial remodeling over time.

Conclusion: Echocardiography is a critical tool for early LVD detection, even in asymptomatic diabetic patients. Findings emphasize routine echocardiographic screening, particularly in those with diabetes duration ≥10 years, poor glycemic control (HbA1c >7%), or elevated hs-CRP levels, recommending assessments at least annually or sooner if symptoms arise. Managing glycemic and lipid profiles, alongside targeted inflammation-reducing strategies, such as anti-inflammatory pharmacologic interventions (e.g., statins, SGLT2 inhibitors, IL-6 inhibitors) and lifestyle modifications (diet, exercise, weight management), is essential for lowering cardiovascular risk. Integrating echocardiographic evaluation into routine diabetic care can help reduce the burden of diabetic cardiomyopathy and improve long-term cardiovascular outcomes.

## Introduction

Diabetes mellitus (DM) is a chronic metabolic disorder affecting millions worldwide, with the prevalence continuing to rise at an alarming rate. The International Diabetes Federation (IDF) estimates that over 537 million adults are living with diabetes globally, a number projected to exceed 783 million by 2045 [1]. DM is associated with various systemic complications, among which cardiovascular diseases (CVDs) remain the leading cause of morbidity and mortality. A significant and often underdiagnosed cardiac manifestation in diabetic patients is left ventricular dysfunction (LVD), a precursor to heart failure and a major contributor to adverse cardiovascular outcomes [2].

LVD encompasses both systolic and diastolic abnormalities, each with distinct pathophysiological implications. Systolic dysfunction, characterized by a reduced ability of the heart to pump blood effectively, is commonly assessed by a decreased left ventricular ejection fraction (LVEF). Diastolic dysfunction involves impaired ventricular relaxation and filling, often present even in the early stages of diabetes [3]. These abnormalities are integral to diabetic cardiomyopathy (DCM), a distinct clinical entity that occurs independently of hypertension or coronary artery disease and reflects the direct impact of diabetes on myocardial structure and function [3,4]. Subclinical diastolic dysfunction can progress to symptomatic heart failure with preserved ejection fraction (HFpEF), while systolic dysfunction leads to heart failure with reduced ejection fraction (HFrEF), underscoring the need for early detection and intervention.

The pathophysiology of LVD in DM is multifactorial, involving hyperglycemia, insulin resistance, systemic inflammation, and oxidative stress. Prolonged hyperglycemia leads to the formation of advanced glycation end products (AGEs), which accumulate in the myocardium, increasing myocardial stiffness and fibrosis. AGEs also bind to their receptors, like receptor for advanced glycation end products (RAGE), on cardiac cells, triggering inflammatory pathways that exacerbate myocardial dysfunction [5]. Insulin resistance and hyperinsulinemia further contribute by impairing myocardial glucose uptake, shifting cardiac metabolism toward fatty acid utilization, and inducing lipotoxicity [6,7]. Systemic inflammation, as indicated by elevated levels of cytokines like interleukin-6 (IL-6) and high-sensitivity C-reactive protein (hs-CRP), plays a critical role in endothelial dysfunction, microvascular rarefaction, and structural myocardial alterations [6-9]. Additionally, autonomic dysfunction, characterized by increased sympathetic activity and reduced vagal tone, contributes to myocardial fibrosis and impaired contractility. Endothelial nitric oxide (NO) imbalance, due to oxidative stress and insulin resistance, further disrupts myocardial perfusion and increases ventricular stiffness, worsening LVD [10].

Emerging research highlights the role of mitochondrial dysfunction and epigenetic modifications in the pathogenesis of diabetic cardiomyopathy. Mitochondrial abnormalities impair ATP production, increase oxidative stress, and promote myocardial apoptosis, leading to progressive cardiac dysfunction. Epigenetic changes, including DNA methylation, histone modifications, and microRNA dysregulation, alter gene expression involved in myocardial fibrosis, inflammation, and metabolism [11,12]. These insights open avenues for novel therapeutic strategies targeting mitochondrial health and epigenetic regulation in diabetic cardiomyopathy.

Echocardiography is crucial for assessing LV function and identifying subclinical abnormalities in diabetic individuals. Conventional echocardiographic parameters, such as LVEF, E/A ratio, E/e′ ratio, and left ventricular mass index (LVMI), provide valuable insights into systolic and diastolic function [13]. Advanced echocardiographic modalities, including tissue Doppler imaging (TDI) and strain analysis, enable the detection of subtle myocardial dysfunction before overt heart failure symptoms emerge [13,14]. However, in India, the accessibility and feasibility of such advanced techniques are limited due to high costs, a lack of trained personnel, and resource constraints. Most healthcare centers rely primarily on basic echocardiographic assessments, potentially delaying early diagnosis and intervention [15].

Numerous studies have highlighted the widespread occurrence of LVD among diabetic individuals [16-18]. Research in Asian populations suggests that up to 40% of diabetic patients exhibit diastolic dysfunction even in the absence of overt heart failure symptoms [17,18]. Studies conducted in Indian and other global populations have demonstrated strong correlations between poor glycemic control, increased inflammatory markers, and left ventricular (LV) abnormalities [19,20]. However, echocardiographic screening practices for LVD remain inadequate in India, primarily due to a lack of awareness, financial limitations, and healthcare disparities. South Asians, particularly Indians, exhibit a higher genetic predisposition to diabetes-related cardiovascular complications, further compounding the disease burden [21]. Additionally, cultural factors often lead to delayed medical consultations, resulting in late-stage diagnosis of LVD. These gaps necessitate a structured approach to integrating echocardiographic screening into routine diabetes management.

This study aims to explore the utility of echocardiography in assessing LVD in individuals with diabetes and to examine the underlying pathophysiological factors contributing to these cardiac abnormalities. By focusing on glycemic control, lipid metabolism, inflammatory markers, and advanced echocardiographic assessment, this research seeks to identify key clinical and biochemical predictors of LVD. The findings will contribute to a better understanding of diabetic cardiomyopathy in the Indian population and inform targeted strategies for early detection, prevention, and management of heart failure in diabetes. Additionally, this study highlights the need for policy changes to enhance echocardiographic accessibility and advocate for routine cardiac screening in diabetic individuals, ultimately aiming to reduce the cardiovascular burden associated with diabetes in India.

## Materials and methods

Study design and setting

This observational cohort study was conducted between January 2021 and December 2023 at Mahatma Gandhi Memorial (MGM) Hospital, Warangal, Telangana, India, which serves a diverse patient population from both urban and rural backgrounds. The hospital is a referral center for a wide range of medical conditions, including diabetes and CVDs, making it an ideal setting for assessing the effectiveness of echocardiography in detecting LVD among diabetic patients. The study site’s large patient pool and diverse demographic representation enhance the generalizability of the findings by capturing a broad spectrum of clinical and biochemical risk factors.

Study population

Inclusion Criteria

The study included adults aged 30 to 70 years with a confirmed diagnosis of type 2 diabetes mellitus (T2DM), based on documented medical records and the American Diabetes Association (ADA) criteria. Diagnosis was established through fasting plasma glucose (≥126 mg/dL), HbA1c (≥6.5%), or two-hour plasma glucose (≥200 mg/dL) during an oral glucose tolerance test. Participants provided written informed consent before enrollment. Both symptomatic (e.g., dyspnea, fatigue) and asymptomatic diabetic patients were included.

Exclusion Criteria

Participants were excluded if they had pre-existing structural heart conditions (e.g., congenital or valvular heart disease), a history of coronary artery disease (CAD) or myocardial infarction, or a previous diagnosis of heart failure. Uncontrolled hypertension was defined as systolic blood pressure (SBP) >180 mmHg and/or diastolic blood pressure (DBP) >110 mmHg and was an exclusion criterion to avoid confounding effects on cardiac function. Additionally, individuals with advanced chronic kidney disease (CKD) at stage 4 or higher (eGFR <30 mL/min/1.73 m^2^) and severe systemic illnesses such as cancer, pregnancy, or lactation were excluded. Pregnant and lactating individuals were excluded due to the physiological and hormonal changes that could influence echocardiographic findings and metabolic parameters. Physical or technical constraints preventing echocardiographic assessment also served as exclusion criteria.

Sample size calculation

The sample size for this study was determined based on the estimated prevalence of LVD in diabetic populations, which is approximately 40% according to previous studies. Using the standard formula for prevalence studies, n = Z² × p × (1 - p) / d², where Z is the Z-score for a 95% confidence level (1.96), p is the estimated prevalence (0.40), and d is the margin of error (0.05), and the calculated sample size was 369 participants [13-15]. To account for potential non-response or dropout rates, an additional 20% was added, resulting in an adjusted sample size of 460 participants. To further enhance statistical power and ensure a representative cohort, the study ultimately enrolled 500 participants. This sample size was considered adequate to yield reliable and generalizable insights into the prevalence and determinants of LVD among diabetic patients.

Data collection

Clinical and Demographic Data

Participants were recruited consecutively from outpatient and inpatient departments. Comprehensive clinical and demographic data, including age, gender, duration of diabetes, smoking and alcohol use, physical activity levels, and medical history, were collected. Details on comorbid conditions, such as hypertension, and current medications were also documented. To ensure consistency and accuracy in data collection, a standardized protocol was followed, with all investigators and healthcare personnel undergoing pre-study training on data entry, quality control, and adherence to clinical guidelines. A structured case record form was used for uniform documentation of patient data. Biochemical sampling was conducted in the fasting state (8-10 AM) after at least eight hours of fasting, and echocardiographic assessments were performed on the same day or within 48 hours to minimize physiological variability. All echocardiographic evaluations were conducted by a trained cardiologist following the American Society of Echocardiography (ASE) guidelines to ensure accuracy and reproducibility.

Biochemical Analysis

Fasting blood samples (5 mL) were obtained from each participant to evaluate essential biochemical markers. Glycemic parameters included fasting blood glucose and HbA1c levels. The lipid profile assessment covered total cholesterol, LDL cholesterol, HDL cholesterol, and triglycerides. Blood glucose and lipid profiles were measured using Beckman Coulter AU5800. HbA1c levels were measured using high-performance liquid chromatography (HPLC) (Bio-Rad D-10 Hemoglobin Testing System, USA). Inflammatory markers, including hs-CRP and interleukin-6 (IL-6), were measured. Renal function was assessed through serum creatinine levels and eGFR. hs-CRP levels were measured using a nephelometric immunoassay (Beckman Coulter IMMAGE 800, USA), ensuring high sensitivity for detecting low levels of systemic inflammation. IL-6 was quantified using a chemiluminescent immunoassay (CLIA) (Siemens ADVIA Centaur XP, Germany), which provides high specificity for pro-inflammatory cytokine detection. All biochemical analyses were conducted in an accredited laboratory following standardized protocols, with strict internal and external quality control measures to ensure accuracy and reproducibility.

Echocardiographic Analysis

Comprehensive two-dimensional echocardiography was performed by a trained cardiologist following American Society of Echocardiography (ASE) guidelines. Systolic function was evaluated by measuring the LVEF, with LVD defined as LVEF <50%. Diastolic function was assessed using the E/A ratio, E/e′ ratio, and left atrial volume index (LAVI). Diastolic dysfunction was defined as an E/A ratio <0.8 with an elevated E/e′ ratio (>14) or an E/A ratio >2 indicating restrictive filling. The LVMI was calculated to detect LV hypertrophy.

TDI was performed to assess myocardial relaxation velocity at the septal and lateral mitral annulus, with e′ velocity <7 cm/s (septal) or <10 cm/s (lateral) considered abnormal. Additionally, two-dimensional speckle tracking echocardiography (2D-STE) was used for global longitudinal strain (GLS) analysis, performed using Philips QLAB software (Philips Medical Systems, USA), with GLS values lower than -18% indicating early myocardial dysfunction.

To minimize variability, all echocardiographic assessments were conducted by a single trained cardiologist, and intra- and inter-observer reliability was assessed in a random subset of 50 patients, with repeat analyses by a second independent cardiologist. The intraclass correlation coefficient exceeded 0.85 for key echocardiographic parameters, ensuring strong reproducibility and consistency in measurements.

Ethical considerations

Ethical clearance for this study was obtained from the Institutional Ethics Committee of Kakatiya Medical College (Approval Number: IEC/KMC/2020/46) on 23rd December 2020. The study adhered to the ethical guidelines of the Indian Council of Medical Research (ICMR), New Delhi. All participants received detailed information regarding the study’s purpose, procedures, potential risks, and benefits, and written informed consent was obtained before inclusion. To ensure participant confidentiality, all data were anonymized using unique coded identifiers, with personal information securely stored separately and accessible only to authorized researchers. Data were maintained in a password-protected database with restricted access. As echocardiography is a non-invasive procedure, potential risks were minimal, and assessments were performed by a trained cardiologist following standard clinical protocols. For venipuncture, strict aseptic techniques were followed, and trained phlebotomists conducted all blood draws to minimize discomfort and reduce the risk of complications. Emergency protocols were in place, but no adverse events were reported. Throughout the study, participant privacy and data security were strictly maintained in compliance with ethical standards.

Statistical analysis

The data were analyzed using IBM SPSS Statistics for Windows, Version 26 (Released 2019; IBM Corp., Armonk, New York, United States). Descriptive statistics were used to summarize the data, with means and standard deviations (SD) reported for continuous variables and frequencies for categorical variables. Inferential analyses included independent t-tests or Mann-Whitney U tests for continuous variables and chi-square or Fisher’s exact tests for categorical variables. Missing data were handled using listwise deletion for critical variables, while multiple imputation (Markov Chain Monte Carlo method) was applied for isolated missing biochemical values (<5%).

Correlations between echocardiographic parameters and biochemical markers were assessed using Pearson or Spearman correlation coefficients. Multivariate logistic regression analysis was performed to identify independent predictors of LVD, with adjustments for age, gender, duration of diabetes, hypertension status, BMI, and lipid profile parameters to minimize confounding effects. Adjusted odds ratios were reported with 95% confidence intervals (CIs). A post hoc power analysis was conducted using G*Power software (version 3.1; Heinrich-Heine-Universität Düsseldorf, Düsseldorf, Germany), confirming a >90% power at α = 0.05 for detecting significant associations between glycemic control (HbA1c levels) and LVD. Statistical significance was set at p < 0.05.

## Results

Distribution of LV function

Participants were classified into normal LV function, diastolic dysfunction, or systolic dysfunction based on echocardiographic parameters following ASE guidelines. Systolic dysfunction was defined as LVEF <50%, while normal LV function was considered LVEF ≥50%. Diastolic dysfunction was identified if at least two of the following criteria were met: E/A ratio <0.8 with E/e′ ratio >14, septal e′ velocity <7 cm/s (or lateral e′ <10 cm/s), or left atrial volume index (LAVI) >34 mL/m²

Among the 500 diabetic participants, 270 (54%) had normal LV function (95% CI: 49.4-58.6), while 140 (28%) were diagnosed with diastolic dysfunction (95% CI: 23.8-32.2) and 90 (18%) had systolic dysfunction (95% CI: 14.7-21.7). The adjusted prevalence rates aligned with these findings. One-way ANOVA revealed a significant difference among LV function categories (F = 18.43, p < 0.001), with an effect size (partial eta-squared) of 0.12, suggesting a moderate association between diabetes and LV dysfunction (Table 1).

**Table 1:** Distribution of LV function among participants Statistical analysis was conducted using one-way ANOVA, yielding an F-value of 18.43 and a p-value of <0.001 (*) in comparison with normal LV function, indicating a statistically significant difference among the groups. Effect size (partial eta-squared) of 0.12 suggests a moderate effect of diabetes on LV function.

LV Function Category	N (%)	95% CI	Adjusted Prevalence (%)
Normal LV Function	270 (54)	(49.4 - 58.6)	54
Diastolic Dysfunction	140 (28)	(23.8 - 32.2)	28
Systolic Dysfunction	90 (18)	(14.7 - 21.7)	18
Total	500 (100)	-	100

Glycemic control and LV function

Poor glycemic control was significantly associated with LV dysfunction. Participants with diastolic and systolic dysfunction exhibited progressively higher HbA1c levels compared to those with normal LV function (p < 0.001, F = 23.67, p < 0.001). The mean HbA1c levels were 7.3% in participants with normal LV function, 8.5% in those with diastolic dysfunction, and 9.2% in those with systolic dysfunction (Table 2). This significant increase in HbA1c levels with worsening LV function reflects the critical role of sustained hyperglycemia in myocardial damage.

**Table 2:** Average HbA1c levels across LV function categories LV: Left Ventricular; HbA1c: Hemoglobin A1c (glycated hemoglobin); CIs: Confidence Intervals HbA1c levels were measured using high-performance liquid chromatography (HPLC) (Bio-Rad D-10 Hemoglobin Testing System, USA)

LV Function Category	Mean HbA1c (%) ± SD	95% CIs	F-value	p-value
Normal LV Function	7.3 ± 0.8	(7.1-7.5)	23.67	<0.001
Diastolic Dysfunction	8.5 ± 0.9	(8.3-8.7)		
Systolic Dysfunction	9.2 ± 1.0	(9.0-9.4)		

Inflammation and LV function

Systemic inflammation, as measured by hs-CRP, was significantly elevated in participants with LVD, with a progressive increase in inflammation corresponding to worsening cardiac function. Participants with systolic dysfunction had the highest hs-CRP levels (4.5 mg/L), followed by those with diastolic dysfunction (3.8 mg/L) and normal LV function (2.1 mg/L) (p < 0.001, F = 19.85) (Table 3). Tukey’s HSD and Bonferroni post hoc tests confirmed significant pairwise differences (p < 0.01) between all LV function groups, reinforcing the role of inflammation in diabetic cardiomyopathy. The activation of nuclear factor-kappa B (NF-κB) and Toll-like receptor (TLR) pathways, triggered by hyperglycemia-induced oxidative stress and AGEs, leads to increased production of IL-6 and tumor necrosis factor-alpha (TNF-α), contributing to myocardial fibrosis, endothelial dysfunction, and impaired ventricular relaxation. Elevated hs-CRP levels reflect heightened hepatic production in response to IL-6 and TNF-α, promoting endothelial dysfunction, arterial stiffness, and myocardial remodeling. These findings highlight systemic inflammation as a key driver of LV dysfunction in diabetic cardiomyopathy, further accelerating microvascular dysfunction, impaired myocardial energetics, and increased ventricular fibrosis.

**Table 3:** Average hs-CRP levels across LV function categories hs-CRP: High-Sensitivity C-Reactive Protein; LV: Left Ventricular; CIs: Confidence Intervals hs-CRP levels were measured using a nephelometric immunoassay

LV Function Category	Mean hs-CRP (mg/L) ± SD	95% CIs	F-value	p-value	Effect Size (Partial Eta-Squared)
Normal LV Function	2.1 ± 0.5	(1.9-2.3)	19.85	<0.001	0.14
Diastolic Dysfunction	3.8 ± 0.6	(3.6-4.0)			
Systolic Dysfunction	4.5 ± 0.7	(4.3-4.7)			

Combined biochemical and functional parameters

**Figure 1:**
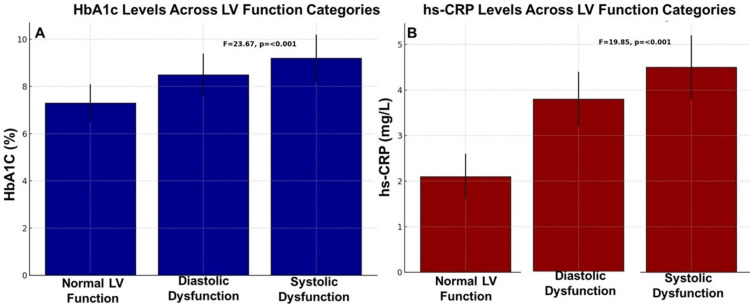
A) HbA1c & B) hs-CRP across LV function in different categories HbA1C: Hemoglobin A1c (Glycated Hemoglobin); hs-CRP: High Sensitive C-Reactive Protein; LV: Left Ventricular

**Figure 2:**
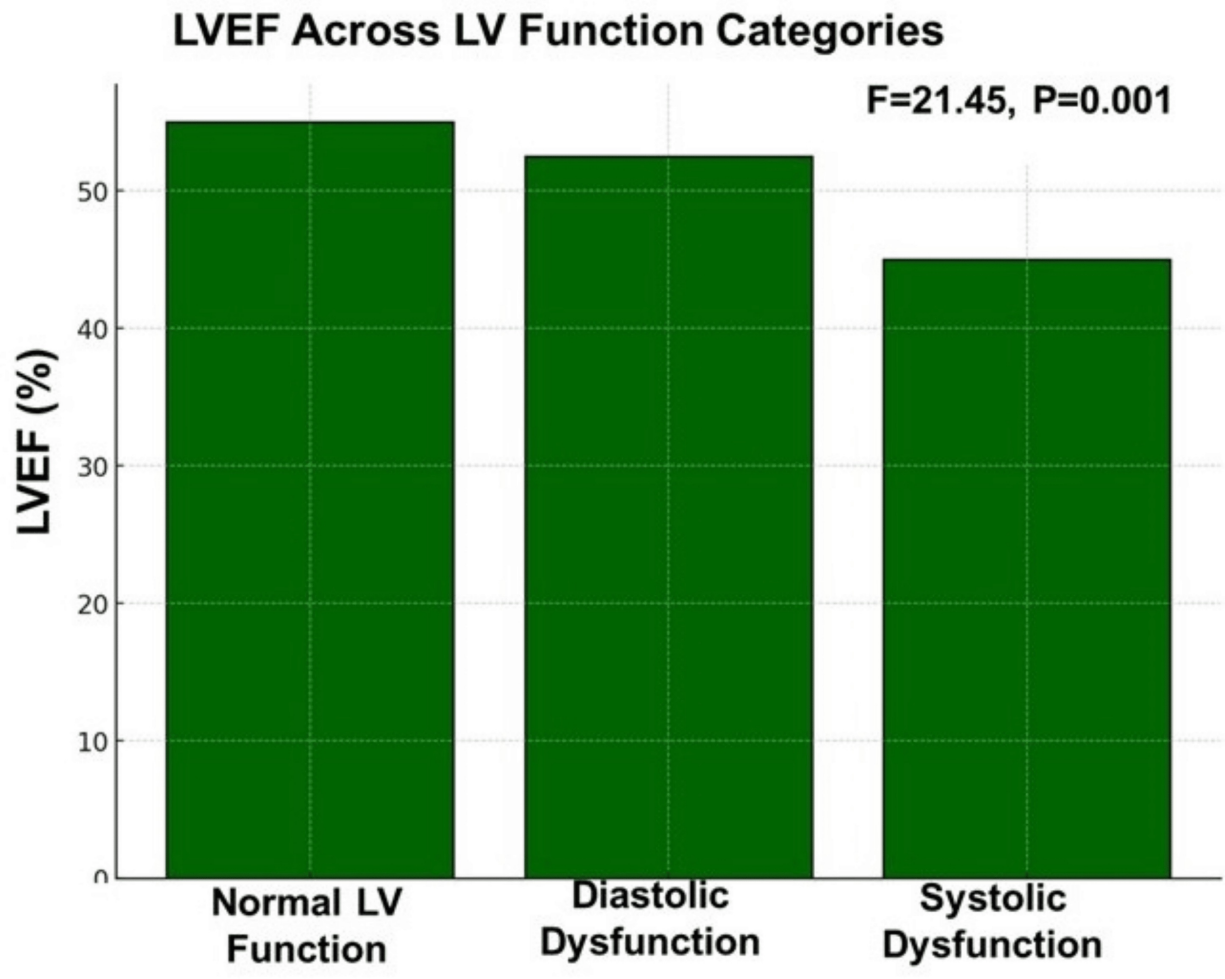
LVEF across LV function categories LV: Left Ventricular; LVEF: Left Ventricular Ejection Fraction

A detailed comparison of key parameters across LV function categories revealed statistically significant differences (p < 0.001 for all variables). HbA1c and hs-CRP levels progressively increased, while LVEF declined with worsening LV function (Figure 1). Participants with normal LV function had LVEF >55%, those with diastolic dysfunction had LVEF between 50 and 55%, and those with systolic dysfunction had LVEF <50% (Figure 2).

Multivariate regression analysis

**Figure 3:**
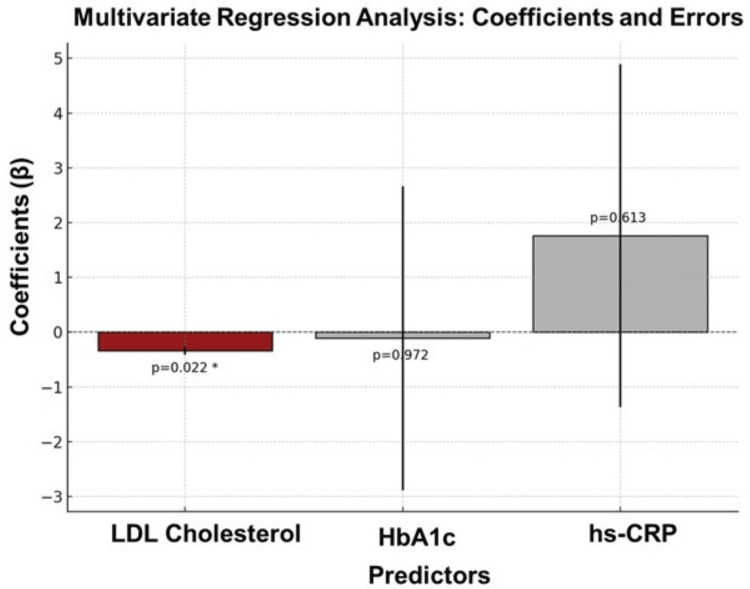
Multivariate regression analysis LDL: Low-Density Lipoproteins; HbA1C: Hemoglobin A1c (Glycated Hemoglobin); hs-CRP: High-Sensitivity C-Reactive Protein Dark red bars represent significant predictors (p < 0.05), while dark gray bars indicate non-significant predictors. p-values are annotated on the bars, with significant values marked by an asterisk (*). A horizontal line at 0 serves as the baseline for the coefficients, providing a reference point for interpretation

This analysis identified LDL cholesterol as a significant predictor of LVEF, with a negative association (β = −0.34, p = 0.022), indicating that higher LDL cholesterol levels are associated with reduced cardiac function. Although HbA1c (p = 0.972) and hs-CRP (p = 0.613) did not show statistically significant relationships with LVEF, this may be attributed to multicollinearity with other covariates (e.g., BMI, diabetes duration, lipid profile), sample size limitations, or a stronger influence on diastolic rather than systolic dysfunction (Figure 3). Despite the lack of statistical significance, the observed trends suggest potential associations that warrant further investigation. The model’s constant (93.44) represents the predicted baseline LVEF when all independent variables are at their reference levels, aligning with the observed mean LVEF in participants with normal LV function (~55-60%), reinforcing the model’s validity. The adjusted R² of 0.32 indicates that approximately 32% of the variance in LVEF can be explained by the included predictors, suggesting moderate explanatory power while highlighting the potential role of other unmeasured factors (e.g., myocardial fibrosis, autonomic dysfunction, genetic predisposition).

## Discussion

This study highlights the significant burden of LVD among diabetic patients, with 270 (54%) of participants exhibiting some form of LVD, including 140 (28%) with diastolic dysfunction and 90 (18%) with systolic dysfunction. These findings align closely with Indian and global studies, reinforcing the interconnected roles of dyslipidemia, hyperglycemia, and systemic inflammation in diabetic cardiomyopathy.

The prevalence of LVD observed in this study is consistent with Indian research, such as the studies by Chaudhary et al. (2018) [15], which reported an LVD prevalence of 36%, highlighting the high cardiac risk in Indian diabetic populations. Globally, studies like the Framingham Heart Study [16] and the China Kadoorie Biobank [17] have demonstrated a 2-4 times higher risk of heart failure among diabetic individuals, emphasizing the universal impact of diabetes on cardiac function.

The current study identified LDL cholesterol as a significant predictor of LVD (β = −0.34, p = 0.022), a finding that aligns with Indian and global data. The ICMR-INDIAB Study (2014) [18] and the study by Anjana et al. (2011) [19] have reported a high prevalence of dyslipidemia among Indian populations, with elevated LDL being a primary risk factor for CVDs. Similarly, the INTERHEART [20] and PURE [21] studies have globally emphasized LDL cholesterol as a key modifiable risk factor for myocardial dysfunction and atherosclerosis. These findings reinforce the need for aggressive lipid management strategies, including statins and PCSK9 inhibitors, to mitigate LVD progression in diabetic individuals.

Although HbA1c did not show a statistically significant association with LVD in this study (p = 0.972), global and Indian studies have established chronic hyperglycemia as a major contributor to myocardial damage. The UKPDS [22] demonstrated a 14% reduction in myocardial infarction risk per 1% decrease in HbA1c, highlighting the importance of tight glycemic control in reducing cardiovascular risk. Similarly, Guria et al. (2022) [23] in India have reported a strong correlation between poor glycemic control (HbA1c >8%) and diastolic dysfunction. The lack of significance in this study may be attributed to sample size limitations, heterogeneity in glycemic control, and potential confounding factors. However, the observed trend underscores the need for stringent glycemic management to prevent diabetic cardiomyopathy.

While hs-CRP was not statistically significant in predicting LVD (p = 0.613) in this study, its role in diabetic cardiovascular dysfunction is well established in the literature. The Ridker et al. (2008) [24] and Ridker et al. (2017) [25] trials have demonstrated that elevated hs-CRP levels are strong predictors of cardiovascular events and atherosclerotic disease progression. Indian studies, such as those by Shrivastava et al. (2015) [26] and Sarangi et al. (2012) [27], have similarly shown a strong association between systemic inflammation and LV dysfunction in diabetic populations. The non-significance in this study may be due to sample size limitations, statin use reducing hs-CRP levels, or variability in inflammatory markers. However, the trend observed suggests that interventions targeting systemic inflammation, such as statins or IL-1β inhibitors, may have potential to improve cardiac outcomes in diabetic patients.

Indian populations are uniquely predisposed to cardiometabolic disorders due to a combination of genetic and lifestyle factors. South Asians have a higher prevalence of small, dense LDL particles, which increases their risk of atherosclerosis and myocardial dysfunction. Additionally, high-carbohydrate diets, sedentary lifestyles, and limited healthcare access contribute to the growing burden of dyslipidemia and diabetes-related CVDs. Tailored interventions addressing dietary modifications, increased physical activity, and early cardiovascular risk screening are essential to combat the rising burden of diabetic cardiomyopathy in India. Successful programs, such as the ICMR-INDIAB study, the Tamil Nadu Diabetes Prevention Program (TNDPP), and the India Heart Watch study, have demonstrated the effectiveness of early lifestyle and pharmacological interventions in improving cardiovascular outcomes in Indian diabetic populations.

Strengths and limitations

This study's strengths include its comprehensive evaluation of LVD using echocardiography, integrating both clinical and biochemical risk factors to enhance diagnostic precision. However, certain limitations should be considered, such as its cross-sectional design, single-center setting, and modest sample size, which may impact the generalizability of the findings and limit the ability to establish causal relationships. Future multicenter, longitudinal studies with larger cohorts are warranted to further validate these findings, assess regional variations in diabetic cardiomyopathy, and explore the long-term impact of glycemic and lipid management on cardiac function.

## Conclusions

Echocardiography emerged as a vital tool for early detection of LVD in diabetic patients, identifying both systolic and diastolic abnormalities, even at subclinical stages. This study highlighted the significant role of dyslipidemia, systemic inflammation, and poor glycemic control in the pathogenesis of LVD. The findings highlight the importance of integrating echocardiographic screening into routine diabetic care to facilitate early diagnosis and targeted interventions. Such strategies, focusing on strict management of glycemic levels and lipid profiles, hold the potential to mitigate cardiovascular risks and improve long-term outcomes for diabetic individuals.
